# Causes and risk factors for same-day discharge failure after total hip and knee arthroplasty: a meta-analysis

**DOI:** 10.1038/s41598-024-63353-9

**Published:** 2024-06-01

**Authors:** José María Lamo-Espinosa, Gonzalo Mariscal, Jorge Gómez-Álvarez, María Benlloch, Mikel San-Julián

**Affiliations:** 1 Hip, Tumors and Pediatric Orthopedic Unit, University Clinic of Navarra, Navarra, Spain; 2 Institute for Research on Musculoskeletal Disorders, Catholic University of Valencia, Carrer de Quevedo, 2, 46001 Valencia, Valencia Spain; 3 Department of Basic Medical Sciences, Catholic University of Valencia, 46001 Valencia, Spain

**Keywords:** Hip arthroplasty, Knee arthroplasty, Joint arthroplasty, Outpatient, Failed discharge, Same-day discharge, Medical research, Risk factors

## Abstract

In recent decades, the trend toward early same-day discharge (SDD) after surgery has dramatically increased. Efforts to develop adequate risk stratification tools to guide decision-making regarding SDD versus prolonged hospitalization after total hip arthroplasty (THA) remain largely incomplete. The purpose of this report is to identify the most frequent causes and risk factors associated with SDD failure in patients undergoing THA and total knee arthroplasty (TKA). A systematic search following PRISMA guidelines of four bibliographic databases was conducted for comparative studies between patients who were successfully discharged on the same day and those who failed. Outcomes of interests were causes and risk factors associated with same-day discharge failure. Odds ratios (OR) were calculated for dichotomous variables, whereas mean differences (MD) were calculated for continuous variables. Meta-analysis was performed using RevMan software. Random effects were used if there was evidence of heterogeneity. Eight studies with 3492 patients were included. The most common cause of SDD failure was orthostatic hypotension, followed by inadequate physical condition, nausea/vomiting, pain, and urinary retention. Female sex was a risk factor for failure (OR 0.77, 95% CI 0.63–0.93), especially in the THA subgroup. ASA score IV (OR 0.33, 95% CI 0.14–0.76) and III (OR 0.72, 95% CI 0.52–0.99) were risk factors, as were having > 2 allergies and smoking patients. General anesthesia increased failure risk (OR 0.58, 95% CI 0.42–0.80), while spinal anesthesia was protective (OR 1.62, 95% CI 1.17–2.24). The direct anterior and posterior approaches showed no significant differences. In conclusion, orthostatic hypotension was the primary cause of SDD failure. Risk factors identified for SDD failure in orthopedic surgery include female sex, ASA III and IV classifications, a higher number of allergies, smoking patients and the use of general anesthesia. These factors can be addressed to enhance SDD outcomes.

## Introduction

Total hip arthroplasty (THA) is a common surgical procedure for the treatment of arthritis, with approximately 7 million Americans currently undergoing hip or knee replacement^1^. The demand for THA is expected to increase substantially in the coming decades owing to an aging population and rising obesity rates^1^. THA has traditionally been associated with a prolonged hospital stay of several days.

In recent decades, there has been a significant trend towards early same-day discharge (SDD) after THA surgery^2^. This shift towards SDD has been driven by various factors, including potential cost savings. For instance, one study estimated savings of approximately $4000 USD per patient using the SDD model compared to traditional post-THA hospitalization^3^.

The literature on THA failure highlights diverse causes, ranging from patient preference to inadequate postoperative motor function and pain control^4,5^. There is a significant variation in the early discharge criteria among institutions, and the definitions of SDD failure are heterogeneous. Consequently, there is uncertainty and lack of consensus regarding the precise risk factors associated with SDD failure after THA, as noted by Lieberman et al. in a recent study^6^. Despite its potential benefits, the practice of SDD after THA remains controversial and has been adopted unevenly across countries and healthcare systems. However, a systematic review reported SDD success rates after THA ranging from 91 to 95%, with significant benefits, such as lower infection risk, greater patient satisfaction, and cost savings^3,7^.

Efforts to develop adequate risk stratification tools to guide the decision-making process between SDD and prolonged hospitalization after THA have remained largely incomplete, as emphasized by Gazendam et al.^8^. Therefore, conducting a meta-analysis to better understand the risk factors associated with SDD failure is imperative for optimizing patient selection and improving clinical outcomes. The objective of this meta-analysis was to synthesize the available evidence and identify the most common causes of SDD failure after THA, and to examine the demographic, clinical, surgical, and anesthetic factors that contribute to a higher risk of SDD failure.

## Methods

### Eligibility criteria

This systematic review was prospectively registered with PROSPERO (CRD42023471109) and conducted according to PRISMA guidelines^9^. A PICOS approach was utilized, where (P) the population included adult patients undergoing total hip arthroplasty, (I) the intervention group comprised patients who were successfully discharged on the same day, (C) the comparison group involved those who failed same-day discharge, and (O) the primary outcomes were causes and risk factors for failure of same-day discharge. Study designs eligible for inclusion were (S) comparative observational studies, such as prospective or retrospective cohort and case–control investigations. Compared same-day versus inpatient discharge rather than focusing on analysis of risk factors associated with ambulatory failure, as this outcome measure did not address the objective of evaluating modifiable risk profiles. Studies involving non-adult populations were excluded as the risk factors and considerations may differ for pediatric populations. Those with incomplete reporting of data were excluded due to the inability to extract pertinent information needed for meta-analysis. Studies using duplicate or overlapping samples were excluded to avoid including non-independent observations and violating statistical assumptions of meta-analysis. Those lacking comparable variables necessary to calculate standardized effect sizes across studies were excluded. Studies with a high risk of bias, were excluded to limit potential skewing of results from low quality evidence.

### Information sources

The systematic search included the following databases: PubMed, EMBASE, Scopus, and the Cochrane Library, covering the period from August 1st to September 30th, 2023. Bibliographic references of included studies were also hand-searched to identify any additional pertinent reports not indexed in the databases. No limits were placed on publication date, study design or language.

### Search methods for identification of studies

The search equation combined (“total hip arthroplasty” OR THA OR “total hip replacement” OR THR OR “total knee arthroplasty” OR TKA OR “total knee replacement” OR TKR) AND “Same-Day Discharge”. Two independent reviewers screened the titles, abstracts, and full texts of potentially eligible studies according to the predefined selection criteria. Any discrepancies in study selection were resolved through discussion between the reviewers.

### Data extraction and data items

Data were extracted from all included studies by two independent reviewers. In the event consensus could not be achieved, involvement of a third reviewer was planned to assess the disputed data items. Baseline study characteristics were collected: study period, region, study type, arthroplasty type (THA or THA/TKA), number of patients, number of females, age, and inclusion criteria. The primary variables were causes of failure and risk factors. The risk factors that could be compared in at least two studies were age, sex, race, BMI, ASA score, approach, previous TJA, general/spinal anesthesia, surgery start time, allergies, and smoking.

### Assessment of risk of bias in included studies

The methodological quality of the included studies was independently assessed by two reviewers using the Methodological Index for Non-Randomized Studies (MINORS) criteria (Supplementary Table S1)^10^.

### Assessment of results

Meta-analysis was performed using the Review Manager 5.4 software package provided by the Cochrane Collaboration. The mean difference (MD) and 95% confidence interval (CI) were calculated for continuous variables, and the odds ratio (OR) for dichotomous variables. Heterogeneity was assessed using the Chi^2^ and I^2^ tests, with I^2^ values ranging from 0 to 100% considered low (25%), moderate (50%), and high (75%) heterogeneity. In the absence of statistical evidence of heterogeneity, a fixed-effects model was employed, whereas a random-effects model was used if significant heterogeneity was detected. To calculate the incidence of the most frequent causes, when the standard error (SE) was not reported, the incidence was calculated using the formula: SE = √I (1 − i)/n and 95% CI = I ± 1.96 × SE; where I = incidence^11^. Pooled incidences with 95% CI were calculated using random- and fixed-effects models. The Cochrane Handbook guidelines were followed to handle the missing data^12^.

### Risk of bias across the studies

A funnel plot analysis was performed using Review Manager 5.4 software to assess potential publication bias graphically. The funnel plot displays each study's standard error on the y-axis against its effect estimate on the x-axis.

### Additional analyses

Subgroup meta-analyses were performed based on arthroplasty type (THA vs TKA), given the review aimed to isolate the THA effect specifically. Subgroups were formed only when at least two studies reported on the outcome within that category.

Sensitivity analyses using Review Manager 5.4 software evaluated the reliability and potential influence of individual studies on outcomes. For each analysis, the single most influential or weighted study was removed based on sample size and the analysis was re-run.

The GRADE (Grading of Recommendations Assessment, Development, and Evaluation) system was used^13^. The GRADE system was applied to each outcome included in our meta-analysis, considering factors such as study design, result consistency, estimation precision, potential biases, and clinical relevance of the findings. GRADEpro, an online platform specifically designed to facilitate implementation of the GRADE system, was used to generate evidence summaries and summary tables.

## Results

### Study selection

**Figure 1:**
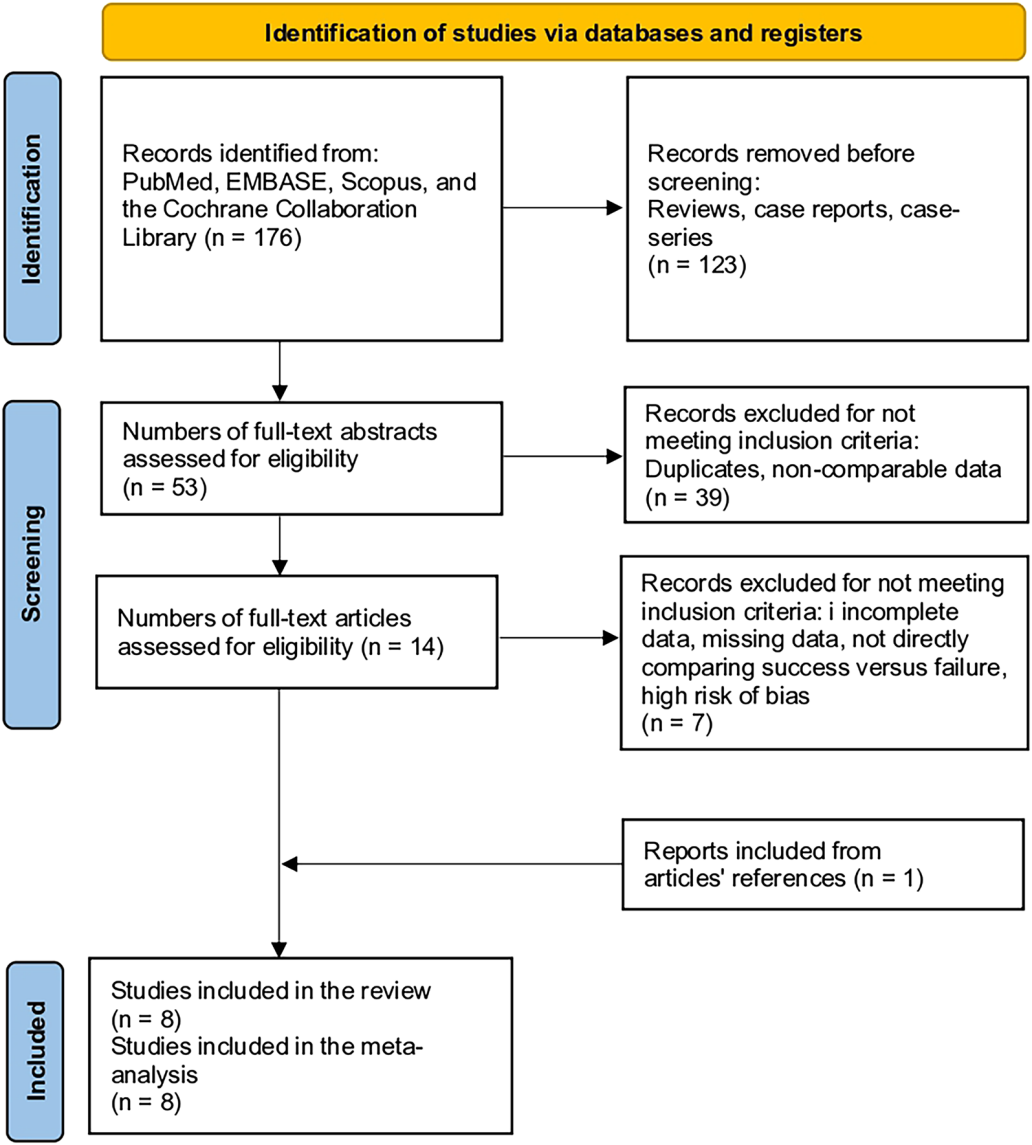
PRISMA flowchart depicting the study selection process for meta-analysis.

The initial literature search yielded 176 results, which were further refined by excluding review articles, case series, and case reports, leaving 53 articles. After reviewing titles and abstracts, duplicate studies, those that were not comparative, and those that did not report relevant outcome variables were excluded, leaving another 39 studies and 14. Upon examining the full text of these articles, a further 7 studies were excluded for not directly comparing success versus failure of same-day discharge, resulting in seven studies eligible for inclusion. The reference lists of the included studies were reviewed, and an two additional study that met the inclusion criteria was identified. In total, eight studies were finally included in the meta-analysis (Fig. 1)^4–6,8,14–17^.

### Study characteristics

Table 1 shows the baseline characteristics of the eight included studies. The included studies were published between 2018 and 2024. Eight studies, including 3492 patients, were included (2904 in the SDD success group and 588 in the SDD failure group). Eight studies were cohort studies (seven retrospective and one prospective). Six of the seven studies were conducted in the USA. A total of 1742 female patients were included (1432 in the SDD success group and 310 in the SDD failure group). Mean age ranged from 55.4 to 64.0 years in the SDD success group and from 56.5 to 64.0 years in the SDD failure group. The inclusion criteria for the individual studies are listed in Supplementary Table S2.

**Table 1:** Main baseline characteristic of the eight included studies.

Study	Period	Region	Type of study	Arthroplasty type	n (Success/Failure)	Female (success/failure)	Age (success/failure)
Foley et al. 2024	2017 to 2020	USA	Retrospective cohort	THA/TKA	(131 (100/31)	78 (61/17)	61.5/62.2
Fraser et al. 2018	NS	USA	Prospective cohort	THA	106 (28/78)	41 (14/27)	57.8/59.1
Gazendam et al. 2023	2019 to 2021	Canada	Retrospective cohort	THA/TKA	527 (426/101)	306 (249/57)	64.0/64.0
Keulen et al. 2020	2013 to 2019	The Netherlands	Retrospective cohort	THA/TKA	525 (415/110)	259 (196/63)	63.0/64.0
Kim et al. 2018	2015 to 2016	USA	Retrospective cohort	THA	163 (143/20)	78 (64/14)	55.4/56.5
Lieberman et al. 2021	2018 to 2020	USA	Retrospective cohort	THA/TKA	271 (226/45)	128 (101/27)	56.6/58.6
Rodriguez et al. 2022	2018 to 2020	USA	Retrospective cohort	THA	278 (182/96)	126 (76/50)	56.8/57.8
Singh et al. 2021	2015 to 2020	USA	Retrospective cohort	THA/TKA	1491 (1384/107)	726 (671/55)	58.0/58.9

*NS* not specified, *TKA* total knee arthroplasty, *THA* total hip arthroplasty.

### Causes of same day discharge failure

The most frequent cause was orthostatic hypotension (Orthostatic hypotension 22.33%, 95% CI 12.66–32.00%; studies = 6), followed by inadequate physical condition (inadequate physical condition 18.17%, 95% CI 17.37–18.97%; studies = 6), nausea and vomiting (nausea and vomiting 11.20%, 95% CI 9.01–13.39%; studies = 5), pain (Pain 11.00%, 95% CI 5.39–16.61%; studies = 5), and urinary retention (Urinary retention 6.00%, 95% CI 1.92–10.08%; studies = 3).

### Risk factors

**Figure 2:**
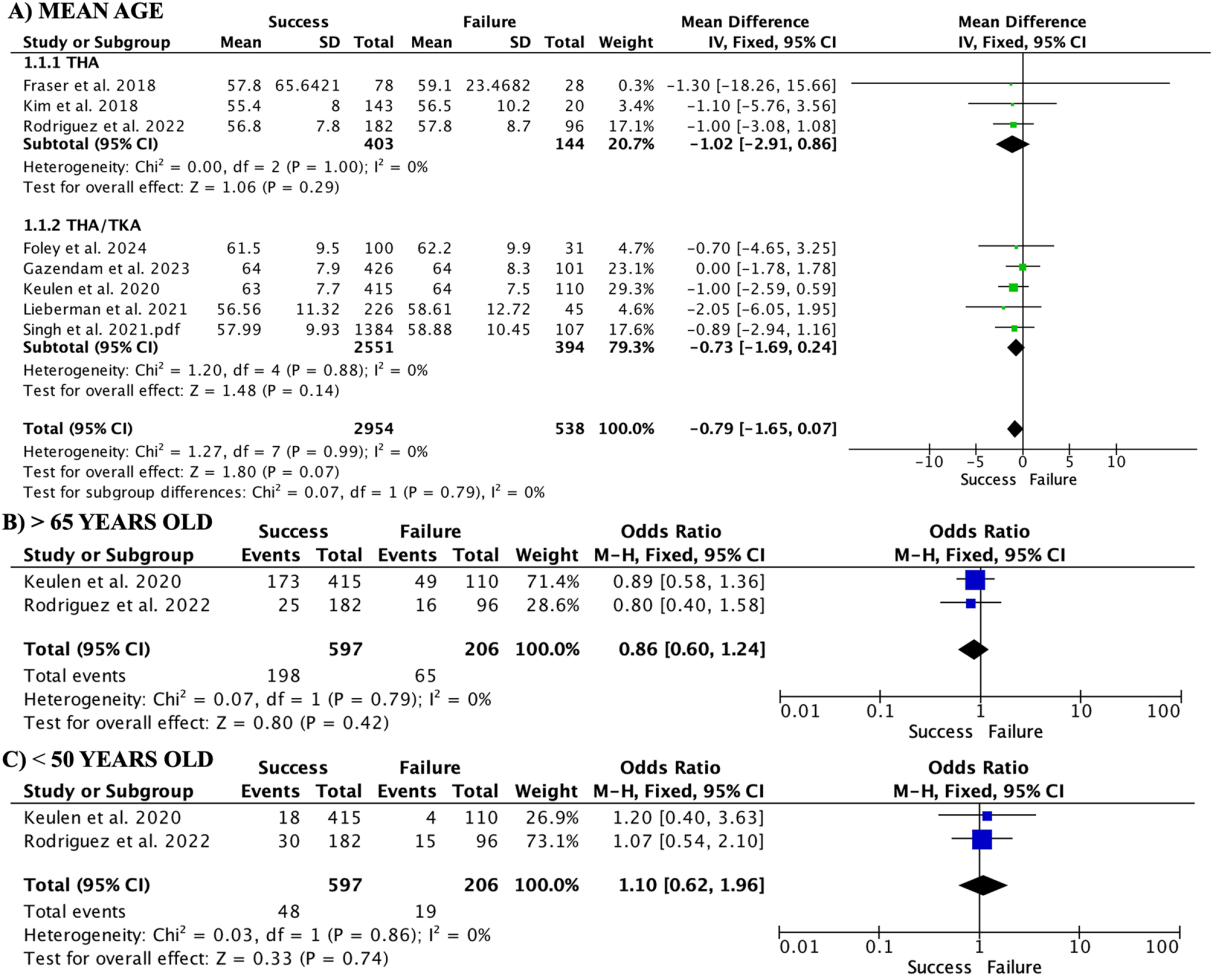
Forest plots showing the effect of age on same-day discharge failure risk. Mean age (**A**) and age over 65 years (**B**) were not associated with discharge failure. No differences were observed in patients under 50 years (**C**).

Regarding risk factors, mean age was not found to be a risk factor (Fig. 2A). When age cutoffs were used, patients older than 65 years did not show a higher risk of failure (Fig. 2B), nor were differences observed in patients under 50 years of age (Fig. 2C). Female sex was associated with a higher risk of SDD failure (OR 0.77, 95% CI 0.63–0.93; participants = 3492; studies = 8; I^2^ = 26%). Subgroups showed that the THA group had significant differences (OR 0.84, 95% CI 0.68–1.04; participants = 2945; studies = 5; I^2^ = 26%), whereas female sex was not a risk factor in the THA/TKA subgroup (OR 0.81, 95% CI 0.65–1.02; participants = 2814; studies = 4; I^2^ = 29%). Race was also not shown to be a risk factor: black (OR 0.59, 95% CI 0.32–1.09; participants = 1654; studies = 2; I^2^ = 0%), Asian (OR 0.56, 95% CI 0.18–1.75; participants = 1654; studies = 2; I^2^ = 0%), and white (OR 1.47, 95% CI 0.95–2.26; participants = 1654; studies = 2; I^2^ = 0%). Mean BMI was also not a risk factor (MD − 0.03, 95% CI − 0.64 to 0.59; participants = 2689; studies = 6; I^2^ = 13%). When a cut-off BMI of 30 (obesity) was used, it also did not appear to be a risk factor (OR 0.90, 95% CI 0.64–1.25; participants = 966; studies = 3; I^2^ = 0%).

**Figure 3:**
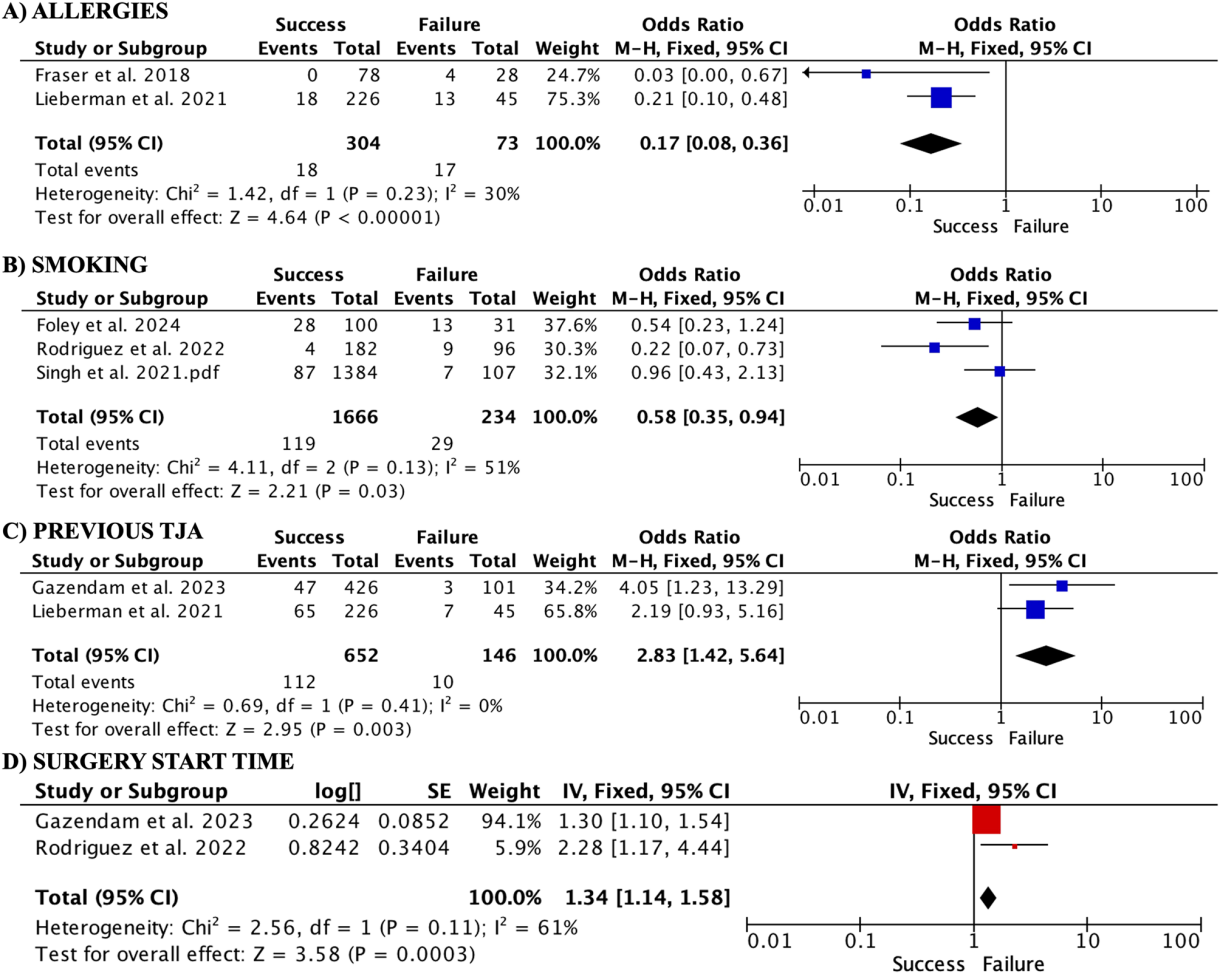
Forest plots of additional risk factors. Having more than 2 allergies was a risk factor for failure (**A**), while previous joint arthroplasty was associated with success (**C**). Earlier surgery time showed a protective effect (**D**). Smoking did not significantly impact outcomes (**B**).

Regarding ASA (Supplementary Table S3), ASA IV showed to be a risk factor for failure (OR 0.33, 95% CI 0.14–0.76). ASA III was also a risk factor (OR 0.72, 95% CI 0.52–0.99). However, when subgroups were analyzed, it was only a risk factor for THA (OR 0.21, 95% CI 0.08–0.55) and not for THA/TKA (OR 0.83, 95% CI 0.59–1.18). ASA II was also a risk factor (OR 0.77, 95% CI 0.43–1.38). ASA I was a factor for SDD success (OR 1.35, 95% CI 1.02–1.78). When the subgroups were analyzed, the THA subgroup did not show differences in ASA I (OR 0.86, 95% CI 0.50–1.47), whereas the THA/TKA subgroup showed ASA I to be a success factor (OR 1.57, 95% CI 1.13–2.18). A number of allergies greater than two were also shown to be risk factors for SDD failure (Fig. 3A). Patients who smoked were at a higher risk of SDD failure (OR 0.58, 95% CI 0.35–0.94) (Fig. 3B). Previous TJA was a success factor (OR 2.83, 95% CI 1.42–5.64; participants, 798; studies, 2; I^2^ = 0%) (Fig. 3C). Earlier surgery time also influenced outcomes, with early time being a success factor (1.34, 95% CI 1.14–1.58; studies = 2) (Fig. 3D).

**Figure 4:**
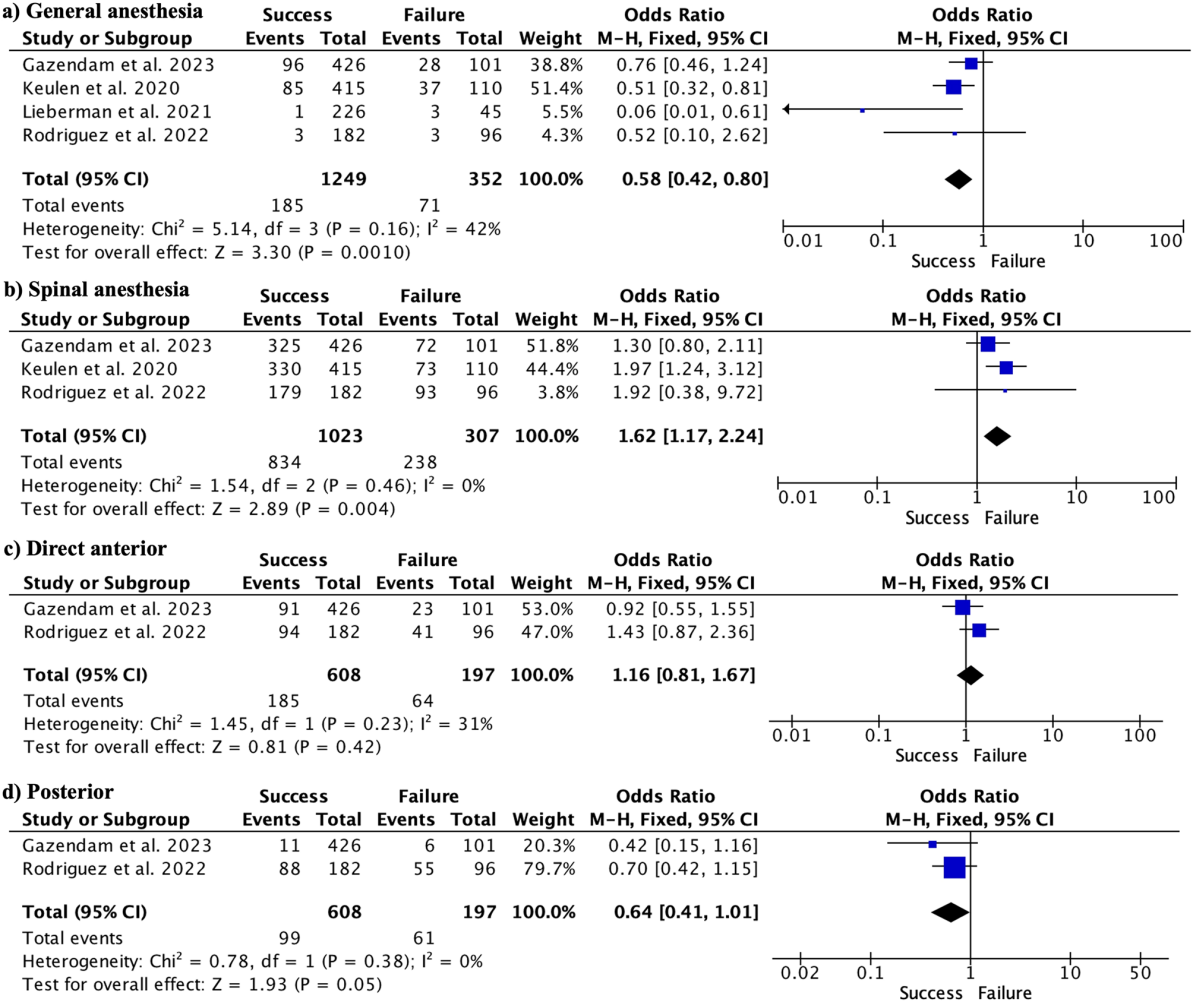
Forest plots showing general anesthesia as a risk factor (**a**), and spinal anesthesia as protective against discharge failure (**b**). The type of approach did not show significant differences neither direct anterior (**c**) nor posterior (**d**) approach.

On the other hand, general anesthesia showed to be a risk factor for SDD failure (Fig. 4a), whereas spinal anesthesia showed to be a factor for SDD success (Fig. 4b). Finally, neither the direct anterior nor posterior approach showed significant differences between the success and failure groups (Fig. 4c,d) respectively.

### Publication bias

Funnel plot analysis revealed symmetry with respect to all analyzed variables (Supplementary Fig. S1), indicating a relatively balanced representation of studies across different levels or categories of these factors and a lower likelihood of publication bias or selective reporting.

### Sensitivity analysis

A sensitivity analysis was performed by eliminating top-weight studies by comparing all outcomes. The direction of the results did not change except for the ASA I and general anesthesia variables, showing no significant differences: (OR 1.00, 95% CI 0.69–1.44; participants = 2836; studies = 7; I^2^ = 0%) and (OR 0.66, 95% CI 0.42–1.03; participants = 1076; studies = 4; I^2^ = 55%) respectively.

### GRADE

The detailed GRADE assessment is shown in Table 2. There was low certainty evidence for the factors of age, sex, BMI, ASA III classification, and spinal anesthesia. However, the results related to general anesthesia and the type of surgical approach showed very low certainty. The studies primarily scored low quality owing to their retrospective design, and indirectness was mainly affected by small differences in demographic data.

**Table 2:** GRADE assessment of the quality of the evidence and the strength of the recommendations.

Certainty assessment							No. of patients		Effect		Certainty	Importance
No. of studies	Study design	Risk of bias	Inconsistency	Indirectness	Imprecision	Other considerations	[Intervention]	[Comparison]	Relative (95% CI)	Absolute (95% CI)		
Age (MD)												
8	Non-randomised studies	Not serious	Not serious	Not serious	Not serious	None	2954	538	–	MD 0.79 lower (1.65 lower to 0.07 higher)	⊕⊕◯◯ Low	IMPORTANT
Female												
8	Non-randomised studies	Not serious	Not serious	Not serious	Not serious	None	1445/2954 (48.9%)	297/538 (55.2%)	OR 0.77 (0.63 to 0.93)	65 fewer per 1000 (from 115 to 18 fewer)	⊕⊕◯◯ Low	IMPORTANT
ASA III												
7	Non-randomised studies	Not serious	Not serious	Not serious	Not serious	None	428/2854 (15.0%)	105/507 (20.7%)	OR 0.72 (0.52 to 0.99)	49 fewer per 1000 (from 88 to 2 fewer)	⊕⊕◯◯ Low	IMPORTANT
General anesthesia												
4	Non-randomised studies	Not serious	Not serious	Serious^a^	Not serious	None	185/1249 (14.8%)	71/352 (20.2%)	OR 0.58 (0.42 to 0.80)	74 fewer per 1000 (from 106 to 34 fewer)	⊕◯◯◯ Very low	CRITICAL
Spinal anesthesia												
3	Non-randomised studies	Not serious	Not serious	Not serious	Not serious	None	834/1023 (81.5%)	238/307 (77.5%)	OR 1.62 (1.17 to 2.24)	73 more per 1000 (from 26 to 110 more)	⊕⊕◯◯ Low	CRITICAL
BMI												
6	Non-randomised studies	Not serious	Not serious	Not serious	Not serious	None	2357	332	–	MD 0.03 lower (0.64 lower to 0.59 higher)	⊕⊕◯◯ Low	CRITICAL
Direct anterior												
2	Non-randomised studies	Not serious	Not serious	Serious^a^	Not serious	None	185/608 (30.4%)	64/197 (32.5%)	OR 1.16 (0.81 to 1.67)	33 more per 1000 (from 44 fewer to 121 more)	⊕◯◯◯ Very low	CRITICAL

^a^Differences in some demographic characteristics; *CI* confidence interval, *MD* mean difference, *OR* odds ratio.

## Discussion

This meta-analysis aimed to identify the causes and risk factors for same-day discharge (SDD) failure after total hip arthroplasty (THA). The most common causes of SDD failure were orthostatic hypotension, inadequate physical condition, nausea and vomiting, pain, and urinary retention. Risk factors for SDD failure included female sex, higher ASA classifications (IV and III), having more than two allergies, smoking patients and general anesthesia. Previous joint replacement surgery, earlier surgery time, and spinal anesthesia were associated with successful SDD. Age, BMI, race, and surgical approach did not significantly impact the outcomes. The included studies in this meta-analysis were of high quality. However, it is important to note that the certainty of the results was low due to various factors, such as limitations in study design and inconsistencies among the included studies. Therefore, caution should be exercised when interpreting the findings.

These factors represent important areas for optimization through individualized perioperative strategies, as discussed in the literature. For example, Fraser et al. suggested limiting the excessive use of opioid narcotics to reduce postoperative nausea and dizziness, which are often exacerbated by medications such as pethidine or tramadol administered for pain control^4^. They proposed prioritizing non-opioid anesthetics and analgesics, such as nonsteroidal anti-inflammatory drugs, acetaminophen, anticonvulsants, such as gabapentin or pregabalin, and local infiltration with long-acting local anesthetics^4^. Other recommended prophylactic measures include alpha-1-adrenergic agonists such as midodrine or ephedrine to increase blood pressure and counteract hypotension as well as a careful strategy of fluids guided by defined hemodynamic goals^4^.

When analyzing the causes of failure between total hip arthroplasty (THA) and total hip/knee arthroplasty (THA/TKA), the causes of failure were found to be similar and did not show significant differences, except in cases of inadequate physical condition. The inadequate physical condition was found to be significantly greater in THA patients than in THA/TKA patients. THA patients may have greater difficulty tolerating physical stress and load associated with surgery and subsequent rehabilitation.

Urinary retention is a common complication associated with the use of urinary catheters and has two important aspects. First, patient mobilization is affected by the presence of a catheter, which limits the patient’s ability to move. This can be particularly problematic in the post-surgical recovery process, where early mobility is crucial for prompt recovery. Additionally, in men with prostate issues, prolonged catheter use may require removal within two or three days. This can be challenging if the patient experiences urinary retention, as it is necessary to insert a new catheter to eliminate retained urine. In this scenario, the recovery process is further delayed, as the patient must wait for the patient to spontaneously urinate after catheter placement. Despite these considerations, Enhanced Recovery After Surgery (ERAS) protocols advocate the avoidance of the routine use of urinary catheters. This is due to the associated risks, such as urinary tract infections, as well as the importance of early patient mobilization for successful recovery. To address this dilemma, strategies have been implemented to balance the need to prevent urinary retention and the risk of catheter use. These strategies include the preoperative assessment of urinary retention risk, optimal pain and fluid management, and selective and temporary catheterization in high-risk patients. Judicious use of local infiltrative anesthetics such as liposomal bupivacaine or ropivacaine could also benefit patients with urinary retention due to prostate hypertrophy or other causes, which is another main cause of SDD failure^18^. Local periarticular infiltration with long-acting drugs has been shown to significantly decrease pain and opioid use during the postoperative period after THA^18^. In addition to providing analgesia, local infiltration prevents systemic side effects of opioids and facilitates functional recovery. Adequate pain control promotes early mobilization and ambulation, which play key roles in achieving a safe same-day postoperative discharge. An inadequate physical condition or acute functional deterioration is the second most frequent cause of SDD failure^4^.

Other proposed strategies are accelerated or “fast-track” rehabilitation protocols, which emphasize early mobilization, preoperative optimization of comorbidities, comprehensive patient education, goal-directed fluid therapy, nausea/vomiting prophylaxis, and multimodal analgesia^19^. Other studies have proposed guidelines for selecting patients with SDD based on specific risk prediction scores. For example, based on the findings of this meta-analysis, Kim et al. instituted a rigorous policy of goal-directed intravenous fluid hydration during the perioperative period^14^. They also incorporated the prophylactic use of antiemetics such as scopolamine in patients with a history of postoperative nausea/vomiting after prior anesthesia, achieving an SDD success rate of 95% through this proactive approach to prevent the two main causes of failure^14^.

These findings could be related to the fact that general anesthesia is associated with a higher risk of SDD failure than neuroaxial or regional anesthesia. While spinal anesthesia may be linked to prolonged recovery from transient motor or sensory impairments, regional blockade promotes earlier rehabilitation by avoiding systemic polypharmacy. The risk of accidentally infiltrating long-acting local anesthetics such as liposomal bupivacaine near the sciatic nerve during the arthroplasty procedure, which can lead to up to three days of transient neurological dysfunction, must also be considered^19^. However, in general, regional anesthesia via lower-extremity nerve blocks allows for faster functional recovery and shorter hospital stay than general anesthesia and intravenous opioid use^19^.

Other identified risk factors included ASA physical status ≥ II, later surgery start time, and history of multiple drug allergies^20,21^. ASA class II or higher is inherently associated with more severe comorbidities, making it difficult to meet the strict criteria for early discharge. Similarly, overweight or obesity with an elevated body mass index did not show an independent association with SDD failure, despite possibly expecting otherwise due to difficulties with early mobilization in these patients.

A later surgery start time likely reflects limited operating room and auxiliary staff availability beyond certain hours, as discussed by Gazendam et al.^8^. Ideally, arthroplasties should be scheduled during times that allow for adequately supervised recovery and rehabilitation, considering the institutional resources. Patients with multiple-drug allergies have worse functional outcomes and higher SDD failure, possibly because of the limited pharmacological options for optimal pain and nausea control in the perioperative period^20,21^.

Protective factors included ASA of Anesthesiologists class I physical status, history of previous joint arthroplasties, and use of neuroaxial versus general anesthesia^6^. Patients with ASA class I enjoyed better overall health without significant comorbidities, thus meeting the criteria for early discharge. Individuals with previous joint surgeries may have more realistic expectations of expected recovery and may be better prepared to undergo the perioperative process both physically and psychologically.

Regarding intrinsic demographic characteristics, a higher risk of SDD failure was observed in women than that in men. This could be related to multifactorial differences in preoperative expectations, pain thresholds, and psychosocial factors between sexes^22^. Women are known to have worse functional outcomes after adjusting for other factors before arthroplasty and worse patient-reported outcome measures (PROMs) after surgery^22,23^. Effective patient communication and structured educational protocols are essential, given that patient expectations are a significant predictor of outcomes in total hip and knee arthroplasty^24^. A recent randomized controlled trial demonstrated that the use of a mobile application improved the quality of life, function, and pain compared to standard education in patients undergoing total knee arthroplasty^25^.

The results of this meta-analysis indicate that among the included studies, neither a direct anterior nor a posterior surgical approach yielded a statistically significant difference in the rates of successful versus failed same-day discharge following total joint arthroplasty. Although direct anterior approaches may offer better functional outcomes and quicker recovery^26^, the data found no differentiation in terms of same-day discharge rates. A possible explanation could be that multiple joint- and patient-specific factors, beyond the surgical approach, play a greater role in determining the feasibility of same-day discharge. For instance, comorbidities, frailty, socioeconomic support at home, and surgical team protocols/resources may influence the discharge timing more than the approach alone. Additionally, the two included studies with 805 combined patients were underpowered to detect small differences in the effect size.

The presence of a caregiver may also influence outcomes as married patients generally receive more support than single, divorced, or widowed individuals. Singh et al. discussed the importance of having a committed caregiver available at discharge to ensure a safe transition home, specifically in the context of enhanced recovery programs^16^. Although not analyzed in this meta-analysis, psychological parameters such as anxiety, depression, and satisfaction strongly impact recovery and adaptation after arthroplasty^27^. Therefore, perioperative psychosocial evaluation and adequate psychological support may optimize SDD outcomes. Dissatisfaction after discharge has been linked to greater opioid use and poorer function^28^.

While costs were not directly compared in this meta-analysis, previous evidence suggests that the SDD model is associated with significant reductions in hospital costs compared with prolonged stays. A study in China reported a significant decrease in total costs following total hip arthroplasty with SDD, saving an average of approximately 10,900 yuan per patient^29^.

All the factors studied in this meta-analysis could be considered and influenced the Enhanced Recovery After Surgery (ERAS) protocol, which is a comprehensive approach designed to optimize the care of surgical patients by implementing evidence-based strategies before, during, and after surgery^30^. It has been observed that only some components of the ERAS program are widely implemented in current clinical practice^30^. This approach aims to minimize surgical stress, maintain physiological function, and accelerate recovery to improve outcomes and reduce hospital stay^30^. ERAS protocols in joint replacement surgery have been associated with a reduced length of hospital stay, decreased pain, cost savings, improved patient-reported outcomes, and decreased incidence of complications, although the quality of evidence remains low^30^. Some of the recommendations of this protocol include preoperative education, optimization of certain factors (smoking, alcohol, anemia), preoperative fasting, standard anesthesia protocols, use of local anesthetics for pain control, prevention of nausea and vomiting, management of perioperative blood loss with tranexamic acid, oral analgesia, maintenance of normothermia, antimicrobial and antithrombotic prophylaxis, perioperative surgical factors, fluid management, postoperative nutritional care, and early mobilization^31^.

### Limitations

This study has several limitations that must be considered when interpreting the results. First, most studies included in this review were retrospective in nature, which can introduce selection bias and limit the data quality. Additionally, owing to the lack of standardization in data collection, there were variables of interest that could not be compared across studies because of the unavailability of that information in published reports. There is a lack of analysis on the role of psychological aspects or possible psychiatric alterations in same-day discharge failure. These factors can significantly influence the outcome, but were not addressed in the studies included in this meta-analysis, which may limit our complete understanding of the determinants of same-day discharge failure after arthroplasty. Furthermore, the low number of articles available for certain variables limited our ability to perform subgroup analyses and to explore potential interactions between variables of interest. This may affect the generalizability of the results and identification of specific risk factors in the determined subpopulations. Another limitation was the inclusion of the results of total hip and knee arthroplasty without proper separation. Although subgroup analyses were performed when possible to control for the heterogeneity between studies, this mixing of results can affect the interpretation and applicability of the findings.

## Conclusions

In conclusion, this study provides valuable information on the causes of failure and risk factors for same-day discharge after arthroplasty. These results highlight the importance of addressing specific identified complications, such as orthostatic hypotension, inadequate physical condition, and nausea/vomiting, to improve the same-day discharge outcomes. Additionally, the female sex, smoking patients, physical status of the ASA of Anesthesiologists, and type of anesthesia were identified as important clinical considerations. The type of approach did not influence the results. These findings underscore the need to implement perioperative management strategies targeted at preventing and adequately addressing identified complications to improve the feasibility and outcomes of same-day discharge after arthroplasty. Additionally, studies that allow for proper separation of the results between total hip arthroplasty (THA) and total knee arthroplasty (TKA) are recommended.

### Supplementary Information

Supplementary Information.

## Data Availability

All data generated or analysed during this study are included in this published article.
