# Supplementary material for: Causes and risk factors for same-day discharge failure after total hip and knee arthroplasty: a meta-analysis
Source: Sci Rep. 2024 Jun 1;14:12627. doi: 10.1038/s41598-024-63353-9 (PMC11144238; doi:10.1038/s41598-024-63353-9)
Supplement: Supplementary file 1 — Supplementary Information. [file 41598_2024_63353_MOESM1_ESM.docx]

**Causes and risk factors for same-day discharge failure after total hip and knee arthroplasty: a meta-analysis**

**José María Lamo-Espinosa, Gonzalo Mariscal, Jorge Gómez-Álvarez, María Benlloch, Mikel San-Julián**


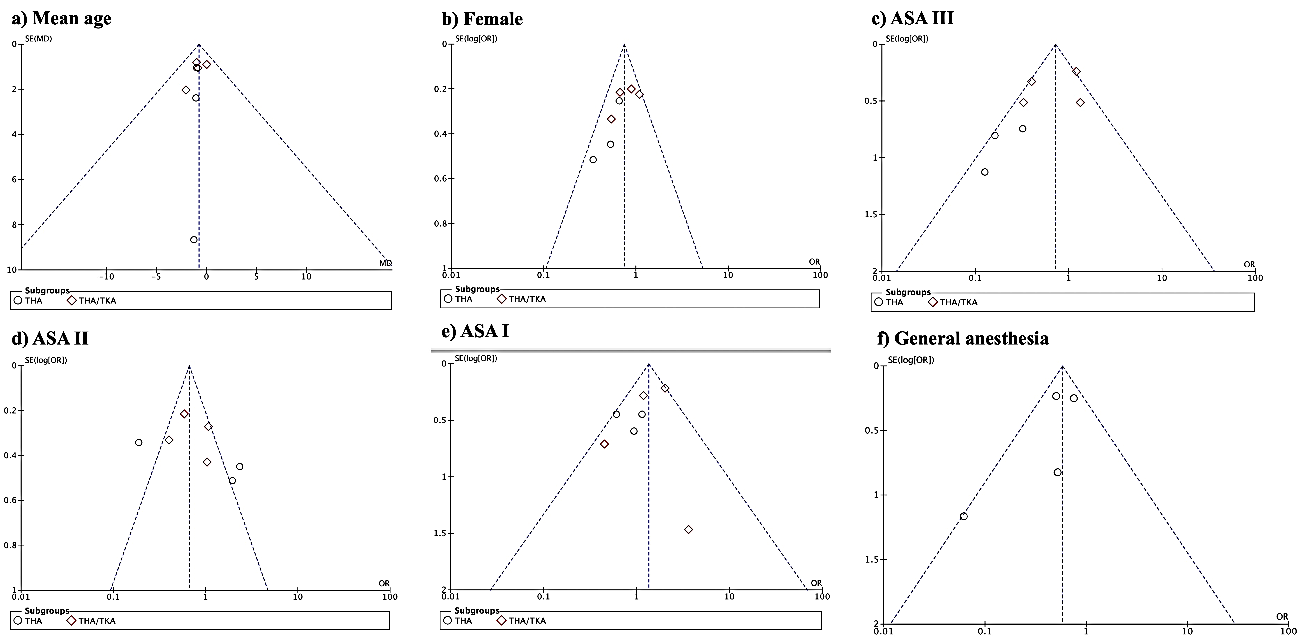


**Supplementary Figure S1.** Funnel plot assessing publication bias. The funnel plot was symmetrical for all variables analyzed.

**Supplementary Table S1.** Assessment of the quality of studies through Methodological Index for Non-Randomized Studies (MINORS).

| **Study** | **Clearly stated aim** | **Consecutive patients** | **Prospective collection data** | **Endpoints** | **Assessment endpoint** | **Follow-up period** | **Loss less than 5%** | **Study size** | **Adequate control group** | **Contemporary group** | **Baseline control** | **Statistical analyses** | **MINORS** |
| --- | --- | --- | --- | --- | --- | --- | --- | --- | --- | --- | --- | --- | --- |
| **Foley et al. 2024** | 2 | 2 | 2 | 2 | 2 | 2 | 2 | 2 | 1 | 2 | 1 | 2 | 22 |
| **Fraser et al. 2018** | 2 | 2 | 2 | 2 | 2 | 2 | 2 | 1 | 2 | 2 | 2 | 2 | 23 |
| **Gazendam et al. 2023** | 2 | 2 | 2 | 2 | 2 | 2 | 2 | 2 | 1 | 2 | 1 | 2 | 22 |
| **Keulen et al. 2020** | 2 | 2 | 0 | 2 | 2 | 2 | 2 | 2 | 1 | 2 | 1 | 2 | 20 |
| **Kim et al. 2018** | 1 | 2 | 0 | 2 | 2 | 2 | 2 | 1 | 2 | 2 | 1 | 2 | 19 |
| **Lieberman et al. 2021** | 2 | 2 | 0 | 2 | 2 | 2 | 2 | 2 | 1 | 2 | 1 | 2 | 20 |
| **Rodriguez et al. 2022** | 2 | 2 | 0 | 2 | 2 | 2 | 2 | 2 | 2 | 2 | 1 | 2 | 21 |
| **Singh et al. 2021** | 2 | 2 | 0 | 2 | 2 | 2 | 2 | 2 | 2 | 2 | 1 | 2 | 21 |

**Supplementary Table S2.** Individualized Inclusion criteria of the seven included studies.

| **Study** | **Inclusion criteria** |
| --- | --- |
| **Foley et al. 2024** | Primary TJA.  Aged 18 years or older. |
| **Fraser et al. 2018** | Primary THA without acute hip fracture or prior hardware needing to be removed.  Unilateral THA.  BMI < 40 kg/m2 304.  Age <75 years old.  Pre-operative Hemoglobin >10g/dL.  No history of cardiopulmonary disease that would necessitate inpatient monitoring after surgery.  No chronic pre-operative opioid use or addiction.  Assistance available at home after discharge.  No other condition or circumstance that would preclude same day discharge.  Subject agrees to same day discharge before surgery. |
| **Gazendam et al. 2023** | Unilateral primary THA or TKA. BMI <40 kg/m2. “Care partner” available to stay with patient for minimum of 48 hours post-op. No history of OSA. Preoperative Hb ⩾120 g/L for females or ⩾130 g/L for males. Live within 30 minutes of hospital with emergency services. Able to communicate in English. |
| **Keulen et al. 2020** | Unilateral joint arthroplasty of the hip (THA) or knee (TKA or UKA).  Patients who were highly motivated and able to be discharged home on the day of surgery (consent was given before planning of surgery).  Postoperative availability of a coach (ie, partner) and adequate social support in the home environment. Relative exclusion criteria were described in Table 1 and aided the surgeon’s discretion in selecting appropriate patients. |
| **Kim et al. 2018** | All patients enrolled in the same-day discharge total hip arthroplasty (THA) program between January 2015 and July 2016 were included in this study. No patient who successfully completed the same-day discharge THA program was excluded from the study. |
| **Lieberman et al. 2021** | Patient interest/expectation. Completed CPAP appointment. Primary joint arthroplasty. Healthy joint coach. Safe discharge location. Established home aftercare visits. |
| **Rodriguez et al. 2022** | Procedures: unilateral primary total knee arthroplasty or simple revisions. Age 18 to 75 years. BMI 18.5 to 37.0 kg/m2. Not currently using warfarin or enoxaparin. Appropiate social support: patient agrees and has a responsible adult to spend the night on the day of discharge. Case scheduled before 12 pm. |
| **Singh et al. 2021** | Patients undergoing bilateral or revision TJA, as well as TJA performed for non-elective or oncologic reasons, and patients who withdrew from the SDD program before the day of surgery were excluded. SDD is defined as patients who were discharged on the same calendar date as their surgery (LOS of 0 days). We defined FTL as any patient with a LOS of 1 night or more. |

**Supplementary Table S3.** Assessment of ASA as a risk factor for same-day discharge failure.

| **Effect size** | **Arthroplasty** | **n studies** | **n participants** | **Fixed effect model (OR 95% CI)** | **I^2^ (%)** | ***P*-Value** |
| --- | --- | --- | --- | --- | --- | --- |
| **ASA I** | Total | 7 | 3361 | OR 1.35, 95% CI 1.02 to 1.78 | 41% | 0.04 |
|  | THA | 3 | 547 | OR 0.86, 95% CI 0.50 to 1.47 | 0% | 0.57 |
|  | THA/TKA | 4 | 2814 | OR 1.57, 95% CI 1.13 to 2.18 | 49% | 0.007 |
| **ASA II** | Total | 7 | 3361 | *OR 0.77, 95% CI 0.43 to 1.38 | 81% | 0.37 |
|  | THA | 3 | 547 | OR 0.93, 95% CI 0.16 to 5.35 | 92% | 0.94 |
|  | THA/TKA | 4 | 2814 | OR 0.70, 95% CI 0.45 to 1.08 | 55% | 0.11 |
| **ASA III** | Total | 7 | 3361 | OR 0.72, 95% CI 0.52 to 0.99 | 67% | 0.001 |
|  | THA | 3 | 547 | (OR 0.21, 95% CI 0.08 to 0.55 | 0% | 0.3 |
|  | THA/TKA | 4 | 2814 | OR 0.83, 95% CI 0.59 to 1.18 | 73% | 0.04 |
| **ASA IV** | Total | 2 | 690 | OR 0.33, 95% CI 0.14 to 0.76 | 0 | 0.009 |

*:Random effec model.
